# Determinants of anemia among patients receiving cancer chemotherapy in Northwest Ethiopia

**DOI:** 10.3389/fmed.2024.1415877

**Published:** 2024-07-11

**Authors:** Samuel Agegnew Wondm, Samuel Berihun Dagnew, Kale Gubae, Tegenu Chanie Tesfaye, Fasil Bayafers Tamene

**Affiliations:** ^1^Clinical Pharmacy Unit, Department of Pharmacy, College of Health Sciences, Debre Markos University, Debre Markos, Ethiopia; ^2^Clinical Pharmacy Unit, Department of Pharmacy, College of Health Sciences, Debre Tabor University, Debre Tabor, Ethiopia; ^3^Pharmacology Unit, Department of Pharmacy, College of Health Sciences, Debre Markos University, Debre Markos, Ethiopia

**Keywords:** cancer, chemotherapy, anemia, determinants, incidence, Northwest Ethiopia

## Abstract

**Background:**

Chemotherapy-induced anemia (CIA) is a hematologic complication that frequently affects patients with cancer undergoing chemotherapy. It is associated with worse treatment outcomes, higher rates of morbidity and mortality, worse quality of life, and higher healthcare costs. The incidence and predictors of CIA in Ethiopia, particularly in Northwest Ethiopian oncology centers, are poorly understood. This study was conducted at Northwest Ethiopian oncology centers to evaluate the incidence and determinants of chemotherapy-induced anemia in adult patients with cancer undergoing chemotherapy.

**Methods:**

This 3-year hospital-based retrospective follow-up study included adult patients with cancer receiving chemotherapy between 2019 and 2021 at two oncology centers in Northwest Ethiopia. Data were collected from October to December 2021. A binary logistic regression model was used to select variables and determine the Crude Odds Ratio (COR). Variables with *P*-value < 0.2 were entered into the multivariable logistic regression and Adjusted odds ratio (AOR) with 95% Confidence intervals (CI) for variables with *P*-value < 0.05 were estimated to show determinants of chemotherapy-induced anemia among cancer patients who received chemotherapy.

**Results:**

A total of 402 patients were included in the final analysis. The overall incidence of CIA was 75.4% (95% CI 70.7, 79.8). Older age [AOR = 1.8, 95% CI (1.4–3.5); *P* = 0.043], hematologic cancer [AOR = 3.7, 95% CI (3.2–5.7), *P* = 0.021], obesity [AOR = 3.4, 95% CI (2.3–6.9); *P* = 0.028], ≥6 chemotherapy cycles [AOR = 3.8, 95% CI (3.2–5.1), *P* = 0.019], cancer metastasis to bone [AOR = 2.9, 95% CI (1.2–4.7), *P* = 0.025] were statistically significant predictors of chemotherapy-induced anemia.

**Conclusion:**

Chemotherapy-induced anemia persisted in a significant percentage of cancer patients. Chemotherapy-induced anemia developed in three-quarters of patients undergoing chemotherapy. Chemotherapy-induced anemia was significantly associated with older age, hematologic malignancy, obesity, a greater number of chemotherapy cycles, and cancer metastasis to bone. To lower the risk of morbidity related to anemia, patients with chemotherapy-induced anemia should be regularly evaluated and treated with appropriate treatment.

## Introduction

Cancer patients frequently experience anemia. The incidence rate ranged from 22.7 (1) to 63% (2) and increased to 89% after treatment (3). Patients undergoing radiation treatment or chemotherapy had a greater prevalence of anemia (4). In addition, the effects of cancer through bone marrow invasion, cancer therapy, such as hormone therapy, targeted therapy, and surgery, as well as the influence of cytokines secreted by cancer cells, all have a role in the increase in anemia incidence among cancer patients (5).

A steady deterioration of cognitive function and energy activity levels is associated with a significant loss in performance status (6). Fatigue, lethargy, dyspnea, anorexia, and difficulty concentrating are symptoms of anemia that can impair a patient's overall functional status and drastically lower adherence to anticancer treatment plans (7). Fatigue caused by anemia may adversely affect a patient's tolerance and willingness to adhere to anticancer treatments; this might affect the dosage and timing of chemotherapy and reduce the effectiveness of treatment (8).

Chemotherapy-induced anemia is associated with reduced effectiveness of radiation, chemotherapy, and chemoradiotherapy, which adversely affects patient treatment outcomes (9, 10) and cancer patients' survival status (11, 12). Cancer-associated anemia incurs psychological and emotional expenses. Furthermore, difficulties such as mental breakdown, dissatisfaction, and lack of drive are also frequently experienced (13).

Chemotherapy-induced anemia varies significantly according to the number of chemotherapy regimens (14), type of chemotherapy (15), type of cancer, and schedule of chemotherapy (16, 17). The type of underlying malignancy, stage of cancer, duration of illness, chemotherapy regimen, and intensity of tumor therapy affect the incidence and severity of chemotherapy-induced anemia (4). Age, gender, platinum compound, type of cancer, and baseline hemoglobin level are significant predictors of chemotherapy-induced anemia (CIA), which makes it possible to predict CIA and take early action to treat anemia and avoid problems (18).

Knowing the determinants and incidence of chemotherapy-induced anemia will aid in determining which groups are most likely to benefit from anemia treatment and improve cancer treatment outcomes. Limited studies have been conducted in Ethiopia on anemia due to cancer (1, 19, 20), without considering cancer treatment-related anemia in current oncology practice. In addition, none of the previous studies assessed the determinants of chemotherapy-induced anemia. Consequently, this multicenter, retrospective follow-up study was conducted to determine the incidence and determinants of chemotherapy-induced anemia among patients with solid and hematologic cancer diagnosed in 2019–2021 receiving chemotherapy in Northwest Ethiopia oncology centers.

## Methods

### Study setting, design, and period

The study was conducted at two large comprehensive and specialized hospitals, Felegehiwot Comprehensive and Specialized Hospital (FHCSH) and the University of Gondar Comprehensive and Specialized Hospital (UoGCSH), Northwest Ethiopia. This retrospective follow-up study was conducted between 2019 and 2021. Data were collected from October to December 2021. The UOGCSH oncology unit had 30 beds, whereas the FHCSH had 20 beds for inpatient cancer treatment.

### Study population, inclusion, and exclusion criteria

The study population comprised all adult patients with a hemoglobin value of ≥12 g/dL and medical records were available during the data collection period. Patients (age ≥ 18 years) with a confirmed diagnosis of malignant tumor, those undergoing first-time cancer treatment, and those with a baseline normal hemoglobin count (≥12 g/dL) were included in the study. Patients who received transfusion within 2 weeks before chemotherapy initiation, those who received radiation therapy within 4 months before chemotherapy initiation, those with baseline anemia, those with incomplete records of hemoglobin levels throughout the chemotherapy cycle, and those with incomplete chemotherapy regimen records were excluded from the study.

#### Sample size determination and sampling technique

**Figure 1:**
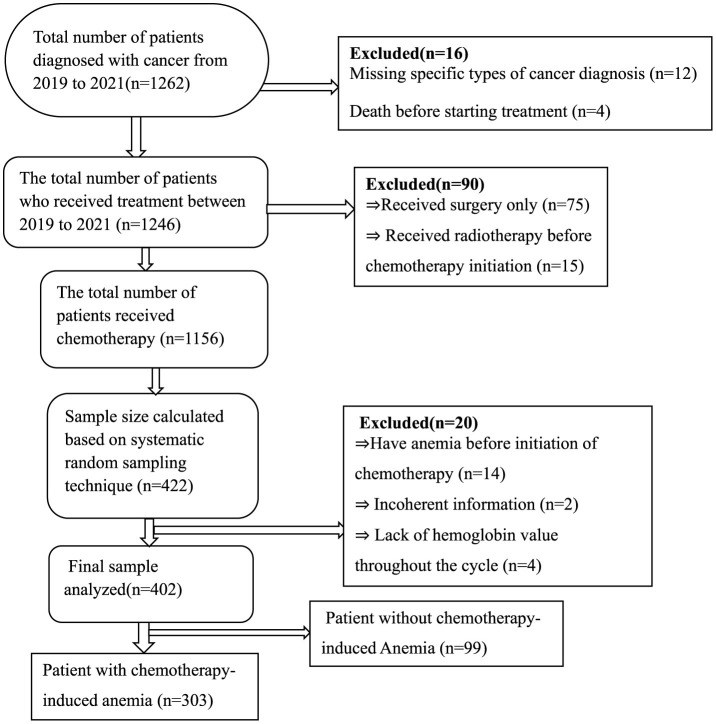
Study population inclusion and exclusion flow diagram.

A Single population formula was used to determine the number of patients required for the study. We used the proportion as 50% in the sample size calculation because no previous comprehensive study on CIA in Ethiopia has been conducted. *n* = p(1-p) x Z2/W2), where n is the desired sample size, Z is the typical normal distribution set at 1.96 (which corresponds to 95% CI), the *p*-value signifies that a positive prevalence was used in calculating sample size, and W is a marginal error (0.05). Then, we computed for *n* = 1.962^*^0.5(1–0.5)/0.052, *n* = 384. After adding a 10% non-response rate, the desired sample size was 422. The proportional allocation of samples to the total population of each hospital was applied using the following formula: n = n × Ni/N, where n = required sample size, Ni = total number of patients at each hospital, and N = total number of patients at selected hospitals. Accordingly, study subjects recruited from UoGCSH with 762 patients = 422^*^762/1,262 = 255 and participants recruited from FHCSH with 500 patients = 422^*^500/1,262 = 167. The total study population from 2019 to 2021 was 1,262, and the sampling interval was 1,262/422 ≈ 3 and we used a systematic random sampling technique for every three patient's medical cards until the required sample size was reached. Finally, 402 patients were considered for the analysis for different reasons (Figure 1).

### Data collection instruments

A pre-tested, semi-structured data abstraction form that was created by reviewing several types of literature was used to extract data from the patient medical charts (3, 21–23). The Common Terminology Criteria for Adverse Events version 5 (CTCAE) was used to quantify leucopenia (yes/no) and thrombocytopenia (yes/no) at any grade (24). The data abstraction format contained information about the patient's sociodemographic characteristics, clinical characteristics, treatment-related characteristics, and laboratory measurements. According to the World Health Organization (WHO) recommendations, body mass index (measured in kilograms per square meter) was classified as underweight (< 18.5), normal weight (18.5–24.9), overweight (25–29.9), and obesity (>30) (25). Participants were divided into two BSA groups: < 2 m^2^ (low) and >2 m^2^ (high). The cutoff point was determined using BSA, which many oncologists use to restrict chemotherapy dosages because of toxicity concerns (26).

### Data quality control measures

To guarantee uniformity, clarity, and convenience of data collection, the questionnaire was pretested on 5% of the sample population at UOGCSH before the data collection period. After the questionnaire underwent minor modifications, real data collection was initiated. Data were collected by four clinical pharmacists after training. A 1-day training session on the goals of the study, types of data collection tools, and ethical issues related to the data collection process was provided to the data collectors. Every day during the data collection period, the completed questionnaire was reviewed to ensure that the data were accurate, clear, and comprehensive.

### Data entry and analysis

Following the questionnaire review, cleaning, and coding, data were entered into Epi-data version 4.6 and exported to STATA version 17 for outcome analysis. The proportion and frequency of categorical variables between the two groups (anemia and no anemia) were determined using the chi-square test. Graphs and tables were used to present the results. To reduce the potential confounding factors of chemotherapy-induced anemia, a binary logistic regression analysis model was employed, and variables with a *p*-value of 0.2 were included in the multivariable logistic regression analysis. Variables with a *p*-value, of < 0.05 with a 95% confidence interval (CI) were considered statistically significant with CIA. The Hosmer-Lemeshow goodness of fit test for logistic regression was performed, and the model was well-fitted to chemotherapy-induced anemia (*P* = 0.45). A contingency coefficient test was performed to determine whether the categorical variables correlated with each other (correlation coefficient = 0.2–0.3). The variance inflation factor was used (VIF = 1.1–4.2) to test whether continuous variables had multicollinearity.

### Outcome measures

A common guideline used in cancer and treatment-related adverse effects, the Common Terminology Criteria for Adverse Events version 5 (CTCAE), defines anemia as follows: mild (Grade 1), hemoglobin (Hg) from 10 g/dL to the lower normal limit; moderate (Grade 2), Hg 8–9.9g/dL; severe (Grade 3), Hg < 8 to 6.5 g/dL; and life-threatening (Grade 4), Hg < 6.5 g/dL (24). Based on the mean corpuscular volume (MCV) and mean corpuscular hemoglobin (MCH), the type of anemia was classified as follows: microcytic anemia (MCV < 80fL), normocytic anemia (80 ≤ MCV ≤ 100fL), macrocytic anemia (MCV>100fL), and hyperchromic anemia (MCH>31pg/cell) (27). Hemoglobin was measured at the beginning of each chemotherapy cycle and continued until the patient received the prescribed medication. Patients who experienced at least one anemia event based on CTCAE throughout each chemotherapy cycle were classified as anemic.

## Results

### Clinical and sociodemographic characteristics of the participants

A total of 402 patients were included in the final analysis. More than half of the patients 236 (58.7%) were female. More than half of the patients 234 (58.2%) were older. More than one-third of patients 140 (34.8%) had comorbidities. More than two-thirds of patients 292 (72.6%) were non-obese. More than half of the patients 226 (56.22%) had a normal body mass index. One-quarter of the patients 101 (25.12%) had a poor performance status. More than one-third of the patients 157 (39.05%) had stage IV cancer. More than half of the patients 220 (54.73%) did not have metastasis. More than one-third of the patients 168 (41.79%) were treated by surgery and chemotherapy. Nearly three-quarters of cases 295 (73.38%) were solid cancer. More than three-quarters of patients 310 (77.1%) received more than six cycles. More than one-third of the patients 152 (37.8%) had leucopenia (Table 1).

**Table 1:** Clinical and sociodemographic characteristics comparison of cancer patients on chemotherapy (*n* = 402).

**Variables**	**Categories**	**Total sample**	**Anemia status**		**Chi-square**
		***n* (%)**	**Anemia**	**No anemia**	***P*-value**
Gender	Male	166 (41.3)	116 (38.3)	50 (50.5)	0.032
	Female	236 (58.7)	187 (61.7)	49 (49.5)	
Age (years)	< 60	168 (41.8)	107 (35.3)	61 (61.6)	0.001
	≥60	234 (58.2)	196 (64.7)	38 (38.4)	
Metastasis status	Lung	56 (13.93)	45 (14.85)	11 (11.1)	0.023
	Liver	41 (10.2)	29 (9.57)	12 (12.12)	
	Bone	58 (14.43)	52 (17.16)	6 (6.06)	
	Lung and bone	14 (3.48)	13 (4.29)	1 (1.01)	
	Liver and lung	13 (3.23)	9 (2.97)	4 (4.04)	
	No distal metastasis	220 (54.73)	155 (51.16)	65 (65.66)	
Comorbidities	Yes	140 (34.8)	101 (33.3)	39 (39.4)	0.27
	No	262 (65.2)	202 (66.7)	60 (60.6)	
BSA (m^2^)	≤ 2	292 (72.6)	207 (68.3)	85 (85.86)	0.001
	>2	110 (27.4)	96 (31.68)	14 (14.14)	
BMI (kg/m^2^)	Underweight	83 (20.65)	83 (23.1)	13 (13.13)	0.12
	Normal	226 (56.22)	161 (53.14)	65 (65.66)	
	Overweight	65 (16.17)	51 (16.83)	14 (14.14)	
	Obesity	28 (6.97)	216 (6.93)	7 (7.07)	
ECOGPS	0	72 (17.91)	41 (13.53)	31 (31.31)	0.02
	I	80 (19.90)	57 (18.81)	23 (23.23)	
	II	105 (26.12)	84 (27.72)	21 (21.21)	
	III	101 (25.12)	85 (28.05)	16 (16.16)	
	IV	44 (10.95)	36 (11.88)	8 (8.08)	
Stage of cancer	I	50 (12.44)	28 (9.24)	22 (22.22)	0.001
	II	80 (19.9)	60 (19.8)	20 (20.2)	
	III	66 (16.42)	54 (17.82)	12 (12.12)
	IV	157 (39.05)	132 (43.56)	25 (25.25)
	Not staged	49 (12.19)	29 (9.57)	20 (20.2)
Treatment goal	Curative	62 (15.4)	53 (17.49)	9 (9.1)	0.56
	Palliative	88 (21.9)	64 (21.12)	24 (24.2)	
	Adjuvant	115 (28.6)	95 (31.35)	20 (20.2)	
	Neoadjuvant	137 (34.1)	91 (30.03)	46 (46.5)	
Cancer medications	< 5	280 (69.65)	214 (70.63)	66 (66.67)	0.74
	5–10	83 (20.65)	61 (20.13)	22 (22.2)	
	≥10	39 (9.7)	28 (9.24)	11 (11.1)	
Type of cancer	Hematologic cancer	107 (26.62)	95 (31.35)	12 (12.12)	0.09
	Solid cancer	295 (73.38)	208 (68.65)	87 (87.88)	
Additional treatment modalities of cancer	Surgery	168 (41.79)	123 (40.59)	45 (45.45)	0.38
	Radiotherapy	53 (13.18)	45 (14.85)	8 (8.08)	
	Radiotherapy +surgery	60 (14.93)	53 (17.49)	7 (7.07)	
Chemotherapy cycles	< 6	92 (22.9)	46 (15.5)	46 (46.5)	0.015
	≥6	310 (77.1)	257 (84.82)	53 (53.5)	
Duration of cancer	≥6 months	284 (70.6)	225 (74.3)	59 (59.6)	0.05
	< 6 months	118 (29.4)	78 (25.7)	40 (40.4)	
Thrombocytopenia	Yes	30 (7.5)	28 (9.2)	2 (2)	0.018
	No	372 (92.5)	275 (90.8)	97 (98)	
Leucopenia	Yes	152 (37.8)	138 (45.5)	14 (14.1)	0.001
	No	250 (62.2)	165 (54.5)	85 (85.9)	

BMI, Body Mass Index; BSA, Body Surface Area; ECOG PS, Eastern Cooperative Oncology Group Performance Status.

### The type of cancer and chemotherapy regimen-related characteristics

A total of 402 patients with cancer who received 21 different chemotherapy regimens were included in the study. Among patients with solid cancer, 125 (31.1%) and 63 (15.7%) patients presented with breast and cervical cancer, respectively. Among patients with hematologic cancer, non-Hodgkin lymphoma 37 (9.2%) and acute lymphoblastic leukemia 25 (6.2%) were frequently encountered (Table 2). Among chemotherapy regimens, adriamycin-cyclophosphamide followed paclitaxel (ACP) 73 (18.2%) and adriamycin-cyclophosphamide (AC) 52 (12.9%) regimens were frequently prescribed for patients with solid cancer, whereas adriamycin, bleomycin, vinblastine, and dacarbazine regimen 20(5%) and cyclophosphamide, doxorubicin, vincristine, and prednisone regimen 20(5%) were the most frequently prescribed chemotherapy for hematologic cancers. The incidence of anemia was common among patients who received AC 46 (15.2), ACP 55 (18.2%), and cisplatin + paclitaxel 58 (19.1%) regimens. However, no significant association was observed between chemotherapy regimens and anemia (Table 3).

**Table 2:** Type of cancer-related characteristics (*n* = 402).

**Variable**	**Category**	**Total sample**	**Anemia status**		**Chi-square**
		***n* (%)**	**Anemia**	**No anemia**	***P*-value**
Solid cancer	Breast cancer	125 (31.1)	92 (30.4)	33 (33.	0.45
	Colorectal cancer	46 (11.4)	20 (6.6)	26 (26.3)	
	Cervical cancer	63 (15.7)	44 (14.5)	19 (19.2)	
	Ovarian cancer	24 (6)	21 (6.9)	3 (3)	
	Lung cancer	19 (4.7)	16 (5.3)	3 (3)	
	GTN	18 (4.5)	15 (5)	3 (3)	
Hematologic cancer	ALL	25 (6.2)	18 (5.9)	7 (7.1)	0.34
	NHL	37 (9.2)	33 (10.9)	4 (4)	
	AML	15 (3.7)	14 (4.6)	1 (1)	
	CLL	6 (1.5)	6 (2)	0 (0)	
	HL	20 (5)	20 (6.6)	0 (0)	
	CML	4 (1)	4 (1.3)	0 (0)	

ALL, Acute Lymphoblastic Leukemia; NHL, Non-Hodgkin lymphoma; AML, Acute Myelogenous Leukemia; CLL, Chronic Lymphocytic Leukemia; Hodgkin Lymphoma; CML, Chronic Myelogenous Leukemia; GTN**;** Gestational trophoblastic disease.

**Table 3:** Type of chemotherapy administered to patients in Northwest Ethiopia oncology centers (*n* = 402).

**Type of chemotherapy regimens**	**Total sample (*n* = 402)**	**Anemia status**		**Chi-square**
	***n* (%)**	**Anemia (*n* = 303)**	**No anemia (*n* = 99)**	***P*-value**
ACP	73 (18.2)	55 (18.2)	18 (18.)	0.32
AC	52 (12.9)	46 (15.2)	6 (6.1)	
CAPOX	3 (0.74)	3 (1)	0 (0)	
Cisplatin and gemcitabine	17 (4.2)	14 (4.5)	3 (3)	
EMACO	16 (4)	15 (5)	1 (1)	
Cisplatin, etoposide and bleomycin	10 (2.5)	7 (2.2)	3 (3)	
Cisplatin and Paclitaxel	74 (18.4)	58 (19.1)	16 (16.3)	
Paclitaxel and carboplatin	12 (3)	11 (3.6)	1 (1)	
FOLFIRI	9 (2.2)	6 (2)	3 (3)	
Methotrexate and leucovorin	2 (0.5)	2 (0.7)	0 (0)	
Carboplatin and Gemcitabine	13 (3.2)	9 (3)	4 (4)	
Cisplatin and 5FU	4 (1)	3 (1)	1 (1)	
Cisplatin and Adriamycin	5 (1.2)	4 (1.3)	1 (1)	
Cisplatin, bleomycin, and 5FU	12 (3)	3 (1)	9 (9.1)	
FOLFOX	18 (4.5)	8 (2.6)	10 (10.1)	
ABVD	20 (5)	15 (5)	5 (5)	
FCR	6 (1.5)	5 (1.7)	1 (1)	
CHOP	20 (5)	11 (3.6)	9 (9.1)	
CHOP-R	17 (4.24)	15 (5)	2 (2)	
CVDOD	15 (3.72)	11 (3.6)	4 (4)	
CMH	4 (1)	2 (0.7)	2 (2)	

AC, Adriamycin-cyclophosphamide; ACP, Adriamycin-cyclophosphamide followed by 4 cycles of paclitaxel; EMACO, Etoposide, Methotrexate, Actinomycin, Cyclophosphamide, and Vincristine; FOLFIRI, Folic acid-fluorouracil -irinotecan; FOLFOX, folic acid- fluorouracil– oxaliplatin; CAPOX, Capecitabine oxaliplatin; 5FU, 5-fluorouracil; ABVD, Adriamycin, Bleomycin, Vinblastine sulfate, and dacarbazine; FCR, Fludarabine, Cyclophosphamide Rituximab; CHOP, Cyclophosphamide, Doxorubicin, Vincristine and Prednisone; CHOPR, Cyclophosphamide, Doxorubicin, Vincristine, Prednisoneand Rituximab; CVDD, Cyclophosphamide, Vincristine, Doxorubicin and Dexamethasone; CMH, Cytarabine, Methotrexate and Hydrocortisone.

### Incidence and severity of chemotherapy-induced anemia

**Figure 2:**
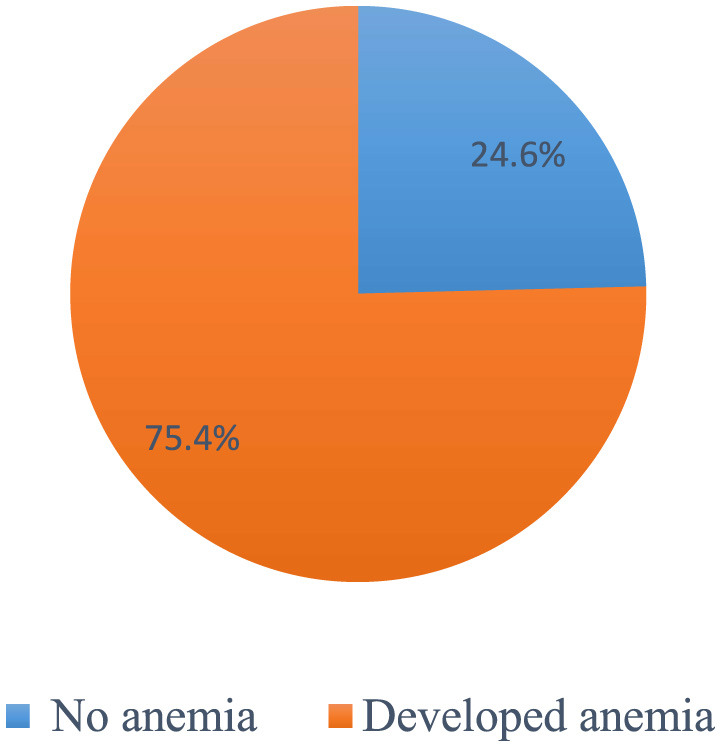
The Overall incidence of chemotherapy-induced amenia among patients with cancer receiving chemotherapy (*n* = 402).

**Figure 3:**
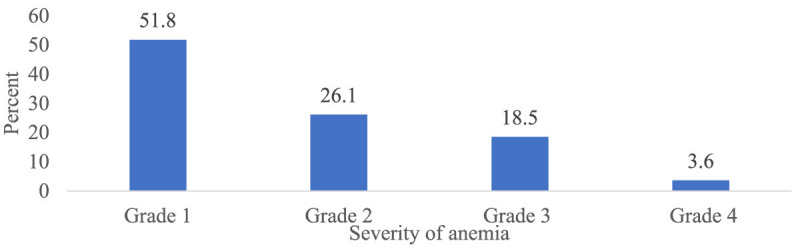
Severity of anemia among patients with cancer receiving chemotherapy (*n* = 303).

**Figure 4:**
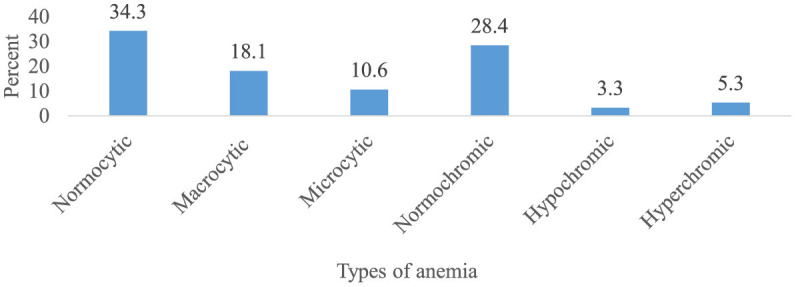
Morphological classification of chemotherapy-induced anemia among cancer patients receiving chemotherapy (*n* = 303).

Overall, 303 patients (75.4 %) (95% CI 70.7, 79.8) had at least one episode of chemotherapy-induced anemia (Figure 2). Among patients who developed anemia, 51.8% (95% CI 46.2, 57.1), 26.1% (95% CI 20.8, 30.7), 18.5% (95% CI 14.2, 23.4), and 3.6% (95% CI 1.7, 6.3) had grade 1, 2, 3, and 4 anemia, respectively (Figure 3). Among all anemic patients, 104 (34.3%) and 86 (28.4%) normocytic and normochromic anemia were frequently identified in the morphological classification of anemia (Figure 4).

### Management of anemia

A total of 3,027 chemotherapy sessions were taken among patients receiving chemotherapy regimens, of which 367 (12.1%) anemia episodes were recorded among 303 patients with treatments of ferrous sulfate (4.3%), ferrous gluconate (3.3%), ferrous fumarate (3.6%), and transfusion (0.9%). The treatment of anemia decreased with increasing chemotherapy courses, with rates of 9 and 7.6% after the first and last chemotherapy cycles, respectively (Table 4).

**Table 4:** Treatment of chemotherapy-induced anemia among cancer patients in Northwest Ethiopia oncology centers.

**Cycle (number of patients)**	**Type of anemia treatment, number of patients (%)**				**Total *n* (%)**
	**Ferrous sulfate**	**Ferrous gluconate**	**Ferrous fumarate**	**Transfusion**	
Chemotherapy cycle 1 (*n* = 402)	14 (3.5)	11 (2.8)	9 (2.2)	2 (0.5)	36 (9)
Chemotherapy cycle 2 (*n* = 402)	17 (4.2)	13 (3.2)	11 (2.7)	5 (1.3)	46 (11.4)
Chemotherapy cycle 3 (*n* = 402)	20 (5)	14 (3.5)	16 (4)	6 (1.5)	56 (14)
Chemotherapy cycle 4 (*n* = 402)	22 (5.5)	19 (4.7)	19 (4.7)	5 (1.2)	65 (16.1)
Chemotherapy cycle 5 (*n* = 381)	20 (5.3)	16 (4.2)	18 (4.7)	4 (1)	58 (15.2)
Chemotherapy cycle 6 (*n* = 366)	18 (4.9)	14 (3.8)	13 (3.6)	3 (0.8)	48 (13.1)
Chemotherapy cycle 7 (*n* = 345)	11 (3.2)	8 (2.3)	12 (3.5)	2 (0.6)	33 (9.6)
Chemotherapy cycle 8 (*n* = 327)	8 (2.4)	6 (1.8)	10 (3.1)	1 (0.3)	25 (7.6)
Total (*n* = 3,027)	130 (4.3)	101 (3.3)	108 (3.6)	28 (0.9)	367 (12.1)

### Determinants of the incidence of chemotherapy-induced anemia

In the univariable logistic regression analysis, 14 variables had a *p*-value of < 0.2 and were candidates for the multivariable logistic regression analysis. In multivariable logistic regression analysis, older age (≥60 years) [AOR = 1.8, 95% CI (1.4–3.5); *P* = 0.043], hematologic cancer [AOR = 3.7, 95% CI (3.2–5.7), *P* = 0.021], obesity [AOR = 3.4, 95% CI (2.3–6.9); *P* = 0.028], ≥6 cycles [AOR = 3.8, 95% CI (3.2–5.1), *P* = 0.019] and metastasis to the bone [AOR = 2.9, 95% CI (1.2–4.7), *P* = 0.025] were significant predictors of CIA (Table 5).

**Table 5:** Bivariable and multivariable logistic regression analysis of chemotherapy-induced anemia among patients with cancer receiving chemotherapy in Northwest Ethiopian oncology centers (*n* = 402).

**Variables**	**Categories**	**CIA (%)**		**COR (95% CI)**	***P*-value**	**AOR (95% CI)**	***P*-value**
		**Yes (*n* = 303)**	**No (*n* = 99)**				
Gender	Female	187	49	1.64 (1.1–2.6)	0.033	2.1(0.5–3.2)	0.076
	Male	116	50	1	Ref	1	Ref
Age	≥60	196	38	2.9 (1.8–4.7)	0.001	1.8 (1.4–3.5)	0.043^*^
	< 60	107	61	1	Ref	1	Ref
Type of cancer	Hematologic cancer	95	12	3.3 (1.7–6.3)	0.09	3.7 (3.2–5.7)	0.021^*^
	Solid cancer	208	87	1	Ref	1	Ref
BSA	>2	14	96	2.8 (1.5–5.2)	0.001	(0.94–3.5)	0.063
	≤ 2	85	207	1	Ref	1	Ref
BMI	Underweight	70	13	2.17 (1.1–4.2)	0.021	1.46 (0.5–3.9)	0.44
	Overweight	51	14	1.47 (0.8–2.8)	0.25	0.43 (0.16–1.1)	0.088
	Obesity	21	7	1.2 (1.1–2.9)	0.07	3.4 (2.3–6.9)	0.028^*^
	Normal	161	65	1	Ref	1	Ref
Stage of cancer	Another state	29	20	1.13 (0.5–2.5)	0.74	1.1 (0.3–3.7)	0.86
	Stage IV	132	25	4.14 (2–8.4)	0.0001	0.99 (0.3–3.4)	0.99
	Stage III	54	12	3.53 (1.5–8.2)	0.003	1.9 (0.6–6.4)	0.28
	Stage II	60	20	2.35 (1.1–5)	0.026	1.14 (0.4–3.4)	0.82
	Stage I	28	22	1	Ref	1	Ref
Albumin	Abnormal	124	18	3.11 (1.8–5.4)	0.02	2.05 (0.87–4.8)	0.097
	Normal	179	81	1	Ref	1	Ref
LDH	Abnormal	155	29	2.53 (1.5–4.1)	0.12	3.7 (0.64–8.2)	0.068
	Normal	148	70	1	Ref	1	Ref
ECOG PS	IV	36	8	3.4 (1.4–8.3)	0.07	2.07 (1.2–4.9)	0.062
	III	85	16	4 (2–8.2)	0.017	3.1 (0.95–5.2)	0.052
	II	84	21	3 (1.6–5.9)	0.001	3.4 (1.2–9.1)	0.071
	I	57	23	1.87 (0.9–3.7)	0.067	2.1 (0.8–5.4)	0.13
	0	41	31	1	Ref	1	Ref
Chemotherapy cycle	≥6	257	53	4.84 (2.9–8)	0.015	3.8 (3.2–5.1)	0.019^*^
	< 6	46	46	1	Ref	1	Ref
Duration of cancer science diagnosis	≥6 month	192	45	2.07 (1.3–3.3)	0.05	2.4 (0.2–4.7)	0.081
	< 6 month	111	54	1	Ref	1	Ref
Metastasis site	Lung	45	11	1.71 (0.83–3.5)	0.14	1.28 (0.4–4.4)	0.68
	Liver	29	12	1.01 (0.5–2.1)	0.97	1.2 (0.3–4.2)	0.81
	Bone	52	6	3.63 (1.5–8.9)	0.005	2.9 (1.2–4.7)	0.025^*^
	Lung and bone	13	1	5.45 (0.69–6.6)	0.11	1.7 (0.1–2.9)	0.69
	Liver and lung	9	4	0.94 (0.28–3.2)	0.92	1.4 (0.2–7.9)	0.73
	Not metastasized	155	65	1	Ref	1	Ref
Thrombocytopenia	Yes	28	2	4.94 (1.2–8.9)	0.031	3.3 (0.2–3.9)	0.087
	No	275	97	1	Ref	1	Ref
Leucopenia	Yes	138	14	5.07 (2.7–9.3)	0.001	2.5 (0.6–6.7)	0.072
	No	165	85	1	Ref		Ref

Ref, reference.

^*^Significance (P < 0.05).

AOR, adjusted odds ratio; CIA, Chemotherapy Induced Anemia; COR, Crude Odds Ratio; CI, Confidence Interval; LDH, Lactate dehydrogenase; BMI, Body Mass Index; BSA, Body Surface area; ECOG PS, eastern cooperative oncology group Performance Status.

## Discussion

Anemia is a frequent hematologic side effect of cancer treatment and is associated with delayed chemotherapy, reduced dosage, and treatment discontinuation. This study aimed to evaluate the determinants of anemia among patients with cancer receiving chemotherapy at Northwest Ethiopia oncology centers.

Anemia was more common in some cancer types than others. Patients with breast cancer (44.3%) and cervical cancer (21.15%) had a higher incidence of CIA. This finding was found to be comparable with that of earlier research demonstrating anemia in patients with gynecologic cancer (1, 3, 19). This finding occurred because individuals receiving gynecological care are more prone to experience vaginal bleeding and becoming anemic during therapy. There was also a significant variation in the incidence of anemia among the chemotherapy regimens. The incidence of anemia was common among patients who received the AC 46 (15.2%), ACP 55 (18.2%), and cisplatin + paclitaxel 58 (19.1%) regimens. Anemia is caused by the increased myelosuppressive effects of platinum, taxane, and anthracycline-based regimens—chemotherapeutic therapies often used for cervical and breast cancer (28). Furthermore, regimens including cisplatin have been shown to affect the digestive system, resulting in anorexia, nausea, and vomiting, which decrease appetite, and they are independent predictors of the incidence and severity of anemia (21). Increased surveillance and a more proactive treatment strategy are needed for those receiving high-risk regimens, such as anthracycline- and carboplatin-taxane-based regimens.

The overall incidence of CIA was 75.4% (95% CI 70.7, 79.8). Comparable results have been obtained in earlier research (29). Our finding was higher than that of a randomized controlled study reporting that CIA was 59% (30). This may be due to the numerous exclusion criteria in RCTs, which may not accurately reflect actual clinical practice. Furthermore, given the use of contemporary oncology procedures, the anemic risk profile could have been altered (24). Our result was higher than those of previous Ethiopian studies that mainly focused on anemia due to malignancy only (1, 19, 29). The reason for this might be that the patients in our research were receiving chemotherapy, which increased the likelihood of anemia after treatment initiation (3).

The incidence of anemia was also higher in Saudi Arabia 44%) (23), Turkey 49.7% (31), Europe 54.4% (29), India 54.7% (32), and China 18% (22). However, our findings were lower than those of a study conducted in California (89.5%) (3) and Saudi Arabia (90.7%) (33). These variations may be attributed to inconsistencies in the definitions and classifications of anemia used across studies. Our finding was also lower than that of a study conducted in China 63.2% (22). This might be because our study included both hematologic and solid cancers, whereas the study in China only included solid cancers. The incidence of cancer is higher in hematologic cancers (up to 90%) (14) than in solid cancers (27, 34–36). This finding implies that the incidence of anemia differs according to the type of cancer.

Old age (≥60 years) was significantly associated with a higher incidence of chemotherapy-induced anemia. This result is comparable with studies conducted in Ethiopia (1), China (21), Sudan (37), and Belgium (38). This might be because aging deteriorates physiological function (39). The reduction of biological function is thought to start at ~45–50 years of age, although the exact age is unknown (40). Fatigue and anemia in cancer patients receiving chemotherapy are frequently caused by disruptions in physiological sleep homeostasis and circadian rhythms (41). According to a prior study, interleukin-6 levels increased with age and were associated with anemia in elderly individuals (42). As a result, erythrocyte synthesis is suppressed by a decline in hematopoietic stem cell reserves and proliferation (43). Compared with younger patients, older individuals have chronic comorbidities, impaired renal function, and generally poor health, all of which contribute to CIA. Elderly people also showed genetic and molecular alterations (44). The elderly are most commonly affected by anemia due to chronic illness, iron deficiency anemia, B12 insufficiency, folate deficiency, gastrointestinal hemorrhage, and myelodysplastic syndrome (45). Consequently, older individuals with cancer-related anemia require more sophisticated, aggressive, and focused therapies.

Hematologic cancer is significantly associated with chemotherapy-induced anemia. This might be the case because unchecked leukocytosis and cytokine production in individuals with hematologic malignancies can have direct and indirect hematologic consequences that lead to CIA in such patients (36). Furthermore, patients with hematologic cancer are more likely to be prescribed multiagent chemotherapy regimens for the treatment of their disease. These drugs have higher toxicities, are longer-lasting, have more intense monitoring requirements, and require hospitalization than palliative-purpose medications. Novel therapies also have distinct and potentially harmful side effects (46).

Obesity was a significant risk factor for chemotherapy-induced anemia in patients with cancer receiving chemotherapy. Our results are in agreement with those of earlier research (36, 47). One explanation could be that obesity decreases the number of nutrients that the gastrointestinal system can absorb, which causes anemia (36). Furthermore, a high-calorie diet inhibits the bone marrow's capacity to produce erythrocytes and promotes excessive production of proinflammatory cytokines (48). Because inflammation prevents the body from storing iron, anemia may occur.

A higher number of chemotherapy cycles (≥6 cycles) were significantly associated with CIA incidence. This finding is in line with earlier research on various tumors undergoing chemotherapy and longer chemotherapy cycles, which are substantial risk factors for anemia following chemotherapy (19, 49, 50). This might be the result of more chemotherapy cycles, which can significantly depress bone marrow through many modes of action (24). Furthermore, the normal life span of human red blood cells, which is ~120 days, maybe the cause of anemia in the fourth cycle (51).

Cancer metastasis to bone is a significant risk factor for chemotherapy-induced anemia. This may be the result of bone marrow suppression caused by cancer metastasis to bone and hemorrhage-induced anemia (52). Cancer metastasizing to bone might be the cause, as this would increase the number of malignant cells and intensify their competition for resources. The patient's hemoglobin levels will drop because of this competition, which will cause anemia. Erythrocytes are also required for angiogenesis resulting from metastasis, which causes anemia caused by chemotherapy (53).

## Strengths and limitations of this study

It is important to evaluate our study's conclusions considering the following limitations in mind. The nutritional status of patients with cancer cannot be assessed. Because of the lack of precise measurement of iron, folate, and B12 status, we were unable to further identify the causes of anemia. Our results may not apply to all patients with cancer in Ethiopia. Despite these limitations, this study has its own strengths. This is the first study to compressively evaluate anemia predictors, anemia severity, and morphological types among patients receiving chemotherapy regimens at Northwest Ethiopia oncology clinics. We recommend a further multicenter prospective cohort study to evaluate the impact of chemotherapy-induced anemia on the treatment outcome of patients with cancer.

## Conclusions and recommendations

The incidence of chemotherapy-induced anemia remains high among patients with cancer receiving chemotherapy. This study found that more than three-quarters of patients who received chemotherapy developed anemia. Old age, hematologic cancer, obesity, a higher number of chemotherapy cycles, and cancer metastasis to bone were significant risk factors for chemotherapy-induced anemia. Patients with chemotherapy-induced anemia should be closely monitored, and a vigilant management strategy should be implemented to reduce the risk of morbidity.

## Data availability statement

The raw data supporting the conclusions of this article will be made available by the authors, without undue reservation.

## Ethics statement

The studies involving humans were approved by Research and Ethics Committee of the Department of Clinical Pharmacy at the University of Gondar. The studies were conducted in accordance with the local legislation and institutional requirements. The Ethics Committee/Institutional Review Board waived the requirement of written informed consent for participation from the participants or the participants' legal guardians/next of kin because Retrospective nature of the study.

## Author contributions

SW: Conceptualization, Data curation, Formal analysis, Investigation, Methodology, Project administration, Resources, Software, Validation, Writing – review & editing. SD: Data curation, Formal analysis, Investigation, Methodology, Resources, Validation, Visualization, Writing – original draft. KG: Data curation, Formal analysis, Investigation, Methodology, Resources, Validation, Visualization, Writing – original draft. TT: Data curation, Formal analysis, Investigation, Methodology, Resources, Software, Validation, Visualization, Writing – review & editing. FT: Data curation, Formal analysis, Methodology, Resources, Software, Supervision, Validation, Visualization, Writing – review & editing.

## References

[1] Kifle E Hussein M Alemu J Tigeneh W. Prevalence of anemia and associated factors among newly diagnosed patients with solid malignancy at Tikur Anbessa Specialized Hospital, Radiotherapy Center, Addis Ababa, Ethiopia. Adv Hematol. (2019) 2019:8279789. 10.1155/2019/8279789 31781226 PMC6855075

[2] Neoh K Stanworth S Pasricha SR Bennett MI. Estimating prevalence of functional iron deficiency anemia in advanced cancer. Support Care Cancer. (2017) 25:1209–14. 10.1007/s00520-016-3511-9 27900547

[3] Xu H Xu L Page JH Cannavale K Sattayapiwat O Rodriguez R. Incidence of anemia in patients diagnosed with solid tumors receiving chemotherapy, 2010-2013. Clin Epidemiol. (2016) 8:61–71. 10.2147/CLEP.S89480 27186078 PMC4847604

[4] Hassan BA Yusoff ZB. Treatment patterns and outcomes in the management of solid cancer patients suffering from anemia in Penang Hospital. Asian Pac J Cancer Prev. (2011) 12:1573–6. 22126501

[5] Aapro M Österborg A Gascón P Ludwig H Beguin Y. Prevalence and management of cancer-related anemia, iron deficiency and the specific role of IV iron. Ann Oncol. (2012) 23:1954–62. 10.1093/annonc/mds112 22575608

[6] Crawford J Cella D Cleeland CS Cremieux PY Demetri GD Sarokhan BJ. Relationship between changes in hemoglobin level and quality of life during chemotherapy in anemic cancer patients receiving epoetin alfa therapy. Cancer. (2002) 95:888–95. 10.1002/cncr.10763 12209734

[7] Ludwig H Strasser K. Symptomatology of anemia. Semin Oncol. (2001) 28(2Suppl.8):7–14. 10.1053/sonc.2001.25391 11395846

[8] Blackwell K Gascón P Sigounas G Jolliffe L. rHuEPO and improved treatment outcomes: potential modes of action. Oncologist. (2004) 9(Suppl.5):41–7. 10.1634/theoncologist.9-90005-41 15591421

[9] Gaspar BL Sharma P Das R. Anemia in malignancies: pathogenetic and diagnostic considerations. Hematology. (2015) 20:18–25. 10.1179/1607845414Y.0000000161 24666207

[10] Zhang LL Zhou GQ Li YY Tang LL Mao YP Lin AH. Combined prognostic value of pretreatment anemia and cervical node necrosis in patients with nasopharyngeal carcinoma receiving intensity-modulated radiotherapy: a large-scale retrospective study. Cancer Med. (2017) 6:2822–31. 10.1002/cam4.1233 29034992 PMC5727247

[11] Birgegård G Gascón P Ludwig H. Evaluation of anemia in patients with multiple myeloma and lymphoma: findings of the European Cancer Anaemia Survey. Eur J Hematol. (2006) 77:378–86. 10.1111/j.1600-0609.2006.00739.x 17044835 PMC1618958

[12] Hasenclever D Diehl V Armitage JO Assouline D Björkholm M Brusamolino E. A prognostic score for advanced Hodgkin's disease. N Engl J Med. (1998) 339:1506–14. 10.1056/NEJM199811193392104 9819449

[13] Candelaria M Cetina L Dueñas-González A. Anemia in cervical cancer patients: implications for iron supplementation therapy. Med Oncol. (2005) 22:161–8. 10.1385/MO:22:2:161 15965279

[14] Cannavale K Xu H Xu L Sattayapiwat O Rodriguez R Bohac C. Epidemiology of chemotherapy-induced anemia in patients with non-Hodgkin lymphoma. Permanente J. (2019) 23:252. 10.7812/TPP/18-252 31314738 PMC6636493

[15] Macciò A Madeddu C Gramignano G Mulas C Tanca L Cherchi MC. The role of inflammation, iron, and nutritional status in cancer-related anemia: results of a large, prospective, observational study. Haematologica. (2015) 100:124. 10.3324/haematol.2014.112813 25239265 PMC4281325

[16] Liu X Qiu H Huang Y Xu D Li W Li Y. Impact of preoperative anemia on outcomes in patients undergoing curative resection for gastric cancer: a single-institution retrospective analysis of 2163 Chinese patients. Cancer Med. (2018) 7:360–9. 10.1002/cam4.1309 29341506 PMC5806112

[17] Lyman GH. Chemotherapy dose intensity and quality cancer care. Oncology. (2006) 20(14Suppl.9):16–25. 17370925

[18] Barrett-Lee PJ Ludwig H Birgegård G Bokemeyer C Gascón P Kosmidis PA. Independent risk factors for anemia in cancer patients receiving chemotherapy: results from the European Cancer Anaemia Survey. Oncology. (2006) 70:34–48. 10.1159/000091675 16493206

[19] Woldemariam A-G Tsehaye A Mokonen W Zeru M Hagos A Tsegay G. Prevalence and Associated Factors of Anemia Among People With Cancer in ACSH, Tigray, Ethiopia. Oakland: The Permanente Press (2023).

[20] Wassie M Aemro A Fentie B. Prevalence and associated factors of baseline anemia among cervical cancer patients in Tikur Anbesa Specialized Hospital, Ethiopia. BMC Women's Health. (2021) 21:1–8. 10.1186/s12905-021-01185-9 33494721 PMC7831239

[21] Men JG Tang J Ding J Chen X Su J-M Liu J-Y. Prevalence and characteristics of anemia in patients with solid cancers at diagnosis in southwest China. Asian Pacific J Cancer Prev. (2011) 12:2825–8. 22393948

[22] Cheng K Zhao F Gao F Dong H Men HT Chen Y. Factors potentially associated with chemotherapy-induced anemia in patients with solid cancers. Asian Pac J Cancer Prev. (2012) 13:5057–61. 10.7314/APJCP.2012.13.10.5057 23244110

[23] Almehmadi M Salih M Elmissbah TE Alsharif A Alsiwiehri N Alzahrani K. Prevalence of anemia among Saudi patients with solid cancers at diagnosis in King Faisal Hospital, Taif Province, Kingdom of Saudi Arabia. PLoS ONE. (2021) 16:e0246202. 10.1371/journal.pone.0246202 33507998 PMC7842985

[24] Groopman JE Itri LM. Chemotherapy-induced anemia in adults: incidence and treatment. J Natl Cancer Inst. (1999) 91:1616–34. 10.1093/jnci/91.19.1616 10511589

[25] Organisation WH. A Healthy Lifestyle-WHO Recommendations. Geneva: Organisation WH (2010).

[26] Griggs JJ Mangu PB Anderson H Balaban EP Dignam JJ Hryniuk WM. Appropriate chemotherapy dosing for obese adult patients with cancer: American Society of Clinical Oncology clinical practice guideline. J Clin Oncol. (2012) 30:1553–61. 10.1200/JCO.2011.39.9436 22473167

[27] Rodgers GM 3rd Becker PS Blinder M Cella D Chanan-Khan A Cleeland C. Cancer- and chemotherapy-induced anemia. J Natl Compr Canc Netw. (2012) 10:628–53. 10.6004/jnccn.2012.0064 22570293

[28] Chaumard N Limat S Villanueva C Nerich V Fagnoni P Bazan F. Incidence and risk factors of anemia in patients with early breast cancer treated by adjuvant chemotherapy. Breast. (2012) 21:464–7. 10.1016/j.breast.2011.10.009 22123411

[29] Ludwig H Van Belle S Barrett-Lee P Birgegård G Bokemeyer C Gascón P. The European Cancer Anaemia Survey (ECAS): a large, multinational, prospective survey defining the prevalence, incidence, and treatment of anemia in cancer patients. Eur J Cancer. (2004) 40:2293–306. 10.1016/j.ejca.2004.06.019 15454256

[30] Cella D Kallich J McDermott A Xu X. The longitudinal relationship of hemoglobin, fatigue, and quality of life in anemic cancer patients: results from five randomized clinical trials. Ann. Oncol. (2004) 15:979–86. 10.1093/annonc/mdh235 15151958

[31] Kenar G Köksoy EB Ürün Y Utkan G. Prevalence, etiology and risk factors of anemia in patients with newly diagnosed cancer. Support Care Cancer. (2020) 28:5235–42. 10.1007/s00520-020-05336-w 32086566

[32] Bahl A Sharma D Basu J Rath G Julka P. Pre-treatment anemia evaluation in Cancer patients attending Radiotherapy Clinic: results from a single Indian Center. Indian J Med Sci. (2008) 62:417–20. 10.4103/0019-5359.44022 19008616

[33] Alghamdi AH Niyaz RI Al-Jifree H Khan MA Alsalmi L. Prevalence of anemia among gynecologic cancer patients who received chemotherapy, radiotherapy, or a combination of both at King Abdulaziz Medical City, Jeddah. Cureus. (2021) 13:e17613. 10.7759/cureus.17613 34646664 PMC8483600

[34] Knight K Wade S Balducci L. Prevalence and outcomes of anemia in cancer: a systematic review of the literature. Am J Med. (2004) 116:11–26. 10.1016/j.amjmed.2003.12.008 15050883

[35] Steegmann J Sanchez Torres J Colomer R Vaz A Lopez J Jalon I. Prevalence and management of anemia in patients with non-myeloid cancer undergoing systemic therapy: a Spanish survey. Clin. Transl. Oncol. (2013) 15:477–83. 10.1007/s12094-012-0953-5 23263906 PMC3663988

[36] Repetto L. Incidence and clinical impact of chemotherapy-induced myelotoxicity in cancer patients: an observational retrospective survey. Crit Rev Oncol Hematol. (2009) 72:170–9. 10.1016/j.critrevonc.2009.03.004 19406660

[37] Hassan FM Weeda EA. Anemia in elderly sudanese lung cancer patients treated with. Open Lung Cancer J. (2010) 3:34–7. 10.2174/1876819901003010034

[38] Verbeke N Beguin Y Wildiers H Canon J Bries G Bosly A. High prevalence of anemia and limited use of therapy in cancer patients: a Belgian survey (Anaemia Day 2008). Support Care Cancer. (2012) 20:23–8. 10.1007/s00520-010-1045-0 21107613

[39] Muthanna FM Karuppannan M Hassan BAR Mohammed AH. Assessment of risk factors associated with anemia severity among breast cancer patients undergoing chemotherapy in Malaysia. Age. (2020) 60:74–2.35464620

[40] Weinert D. Age-dependent changes of the circadian system. Chronobiol Int. (2000) 17:261–83. 10.1081/CBI-100101048 10841207

[41] Dawson D Ian Noy Y Härmä M Akerstedt T Belenky G. Modelling fatigue and the use of fatigue models in work settings. Accid Anal Prev. (2011) 43:549–64. 10.1016/j.aap.2009.12.030 21130216

[42] Joosten E Pelemans W Hiele M Noyen J Verhaeghe R Boogaerts MA. Prevalence and causes of anemia in a geriatric hospitalized population. Gerontology. (1992) 38:111–7. 10.1159/000213315 1612458

[43] Kim Y-J Han KD Cho K-H Kim Y-H Park Y-G. Anemia and health-related quality of life in South Korea: data from the Korean national health and nutrition examination survey 2008-2016. BMC Public Health. (2019) 19:735. 10.1186/s12889-019-6930-y 31196013 PMC6567528

[44] Hung N Shen C-C Hu Y-W Hu L-Y Yeh C-M Teng C-J. Risk of cancer in patients with iron deficiency anemia: a nationwide population-based study. PLoS ONE. (2015) 10:e0119647. 10.1371/journal.pone.0119647 25781632 PMC4363660

[45] Smith DL. Anemia in the elderly. Am Fam Physician. (2000) 62:1565–72. 11037074

[46] Brudno JN Kochenderfer JN. Recent advances in CAR T-cell toxicity: mechanisms, manifestations, and management. Blood Rev. (2019) 34:45–55. 10.1016/j.blre.2018.11.002 30528964 PMC6628697

[47] Aigner E Feldman A Datz C. Obesity as an emerging risk factor for iron deficiency. Nutrients. (2014) 6:3587–600. 10.3390/nu6093587 25215659 PMC4179177

[48] Camaschella C. New insights into iron deficiency and iron deficiency anemia. Blood Rev. (2017) 31:225–33. 10.1016/j.blre.2017.02.004 28216263

[49] Badheeb AM Ahmed F Badheeb MA Obied HY Seada IA Al Jumman A. Anemia profiles in cancer patients: prevalence, contributing factors, and insights from a retrospective study at a Single Cancer Center in Saudi Arabia. Cureus. (2023) 15:e42400. 10.7759/cureus.42400 37621805 PMC10446849

[50] Muthanna F Karuppannan M Abdulrahman E Uitrakul S Rasool BAH Mohammed AH. Prevalence and associated factors of anemia among breast cancer patients undergoing chemotherapy: a prospective study. Adv Pharmacol. Pharmaceut. Sci. (2022) 2022:v1. 10.21203/rs.3.rs-1459914/v1 35464620 PMC9023199

[51] Shemin D Rittenberg D. The life span of the human red blood cell. J Biol Chem. (1946) 166:627–36. 10.1016/S0021-9258(17)35201-8 20276177

[52] Lim S Lee C-M Park J-M Jung S-Y Lee K-B. An association between preoperative anemia and poor prognostic factors and decreased survival in early-stage cervical cancer patients. Obstet Gynecol Sci. (2014) 57:471–7. 10.5468/ogs.2014.57.6.471 25469335 PMC4245340

[53] Zhao L He R Long H Guo B Jia Q Qin D. Late-stage tumors induce anemia and immunosuppressive extramedullary erythroid progenitor cells. Nat Med. (2018) 24:1536–44. 10.1038/s41591-018-0205-5 30297899 PMC6211844

